# Diurnal Variation and Spatial Distribution Effects on Sulfur Speciation in Aerosol Samples as Assessed by X-Ray Absorption Near-Edge Structure (XANES)

**DOI:** 10.1155/2012/696080

**Published:** 2012-09-05

**Authors:** Siwatt Pongpiachan, Kanjana Thumanu, Warangkana Na Pattalung, Phoosak Hirunyatrakul, Itthipon Kittikoon, Kin Fai Ho, Junji Cao

**Affiliations:** ^1^Center for Research & Development of Disaster Prevention & Management, School of Social and Environmental Development, National Institute of Development Administration (NIDA), 118 Moo 3, Sereethai Road, Klong-Chan, Bangkapi, Bangkok 10240, Thailand; ^2^SKLLQG, Institute of Earth Environment, Chinese Academy of Sciences (IEECAS), No. 10 Fenghui South Road, P.O. Box 17, Xi'an 710075, China; ^3^Synchrotron Light Research Institute, 111 University Avenue, P.O. Box 93, Nakhon Ratchasima 30000, Thailand; ^4^Bara Scientific Co., Ltd. (Head Office), U Chu Liang Building Floor 7, 968 Rama 4 Road Silom, Bangrak, Bangkok 10500, Thailand

## Abstract

This paper focuses on providing new results relating to the impacts of *Diurnal variation*, *Vertical distribution*, and *Emission source* on sulfur K-edge XANES spectrum of aerosol samples. All aerosol samples used in the diurnal variation experiment were preserved using anoxic preservation stainless cylinders (APSCs) and pressure-controlled glove boxes (PCGBs), which were specially designed to prevent oxidation of the sulfur states in PM_10_. Further investigation of sulfur K-edge XANES spectra revealed that PM_10_ samples were dominated by S(VI), even when preserved in anoxic conditions. The “Emission source effect” on the sulfur oxidation state of PM_10_ was examined by comparing sulfur K-edge XANES spectra collected from various emission sources in southern Thailand, while “Vertical distribution effects” on the sulfur oxidation state of PM_10_ were made with samples collected from three different altitudes from rooftops of the highest buildings in three major cities in Thailand. The analytical results have demonstrated that neither “Emission source” nor “Vertical distribution” appreciably contribute to the characteristic fingerprint of sulfur K-edge XANES spectrum in PM_10_.

## 1. Introduction

Sulfur speciation in aerosol particles plays a crucial role in both Earth's energy balance and acid deposition [1–5]. Numerous efforts have been made to deepen insight into and clarify the oxidation pathways of sulfate in aerosol samples [6, 7]. There is a significant precedent in the use of X-ray absorption near edge structures (XANES) to assess the presence of sulfur in particulate matter [8]. Over the past decade, several reference publications have indicated that the most stable oxidation state of sulfur in aerosol is +6 (S(VI)) [9–12]. These findings were supported by recent experimental data suggesting that (NH_4_)_2_SO_4_ and CaSO_4_-2(H_2_O) are dominant sulfur species in aerosols [13]. Despite of these facts, there are several outstanding questions and information gaps which merit additional study for clarification. Such is the case for investigating the role of emission source, diurnal variation, and vertical distribution on sulfur oxidation states and on sulfur K-edge XANES spectra in PM_10_. Since the conversion of gaseous SO_2_ to particulate SO_4_^−2^ can dramatically alter the sulfur oxidation state, all ambient sulfur species are required to preserve in anoxic condition due to its relatively high oxidation rate as previously discussed in several publications [14–16].

Despite the spatially dynamic movement of aerosols, the majority of studies so far have concentrated only on sulfur speciation as a result of particle size segregation at ground level. Up to now, research was focused predominantly on defining sulfur K-edge XANES spectrum of aerosol samples collected from monitoring sites classified either Urban or remote/rural. Furthermore, the samples have always been collected as close to the source as possible, while measurement of sulfur oxidation states in samples taken from sites *adjacent* to the emission sources have been largely neglected. Neither have there been studies examining sulfur speciation in PM_10_ as a function of altitude. The answer of how *emission source and proximity* and *vertical distribution* affect sulfur oxidation states in aerosols offers crucial insight for understanding aerosol behavior and pollutant transport, with ultimate implications for mitigation and policy. Thus, the overall aims of this study are

1. to qualitatively investigate the impact of diurnal variation on sulfur K-edge XANES spectra of PM_10_ collected at ground level,
2. to analyze sulfur K-edge XANES spectra in PM_10_ samples collected adjacent to various emission sources representative of biomass burning, crude oil combustion, diesel emission, timber burning and traffic exhaust in Songkla Province, southern Thailand, and
3. to conduct a qualitative investigation of the “altitude effect” on sulfur K-edge XANES spectra in PM_10_ samples collected at urban sites throughout Bangkok, Chiang-Mai and Hat-Yai, Thailand.

## 2. Materials and Methods

### 2.1. Sample Collection and Sampling Site Descriptions

#### 2.1.1. Sulfur K-Edge XANES Experiment

**Figure 1:**
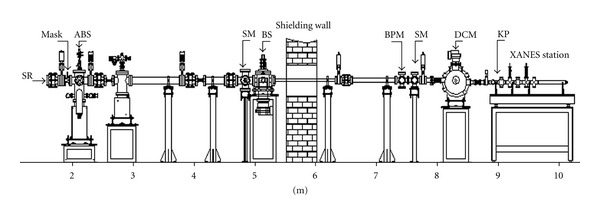
Schematic view of beamline no. 8. Polychromatic Synchrotron X-rays (SRs) are emitted by a bending magnet (not shown) transmitted through the main components of the beamline, namely, the mask, heat absorber (ABS), screen monitor (SM), beam shutter (BS), beam position monitor (BPM), double crystal monochromator (DCM), and polyimide window (KP). Distance from the bending magnet source to each component is given on the scale below the diagram.

**Figure 2:**
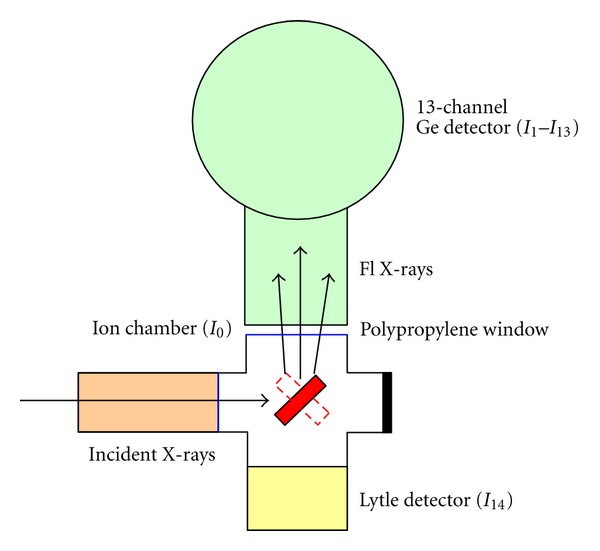
Apparatus setup for fluorescence-mode XANES. Absorbance in XANES spectra is given by *I*_*n*_/*I*_0_.

XANES was performed on the samples, and standards were measured at beam line no. 8 in the Siam Photon Laboratory [17]. Photon energy from an X-ray beam transported through the beam line was scanned by InSb(111) double crystals installed in the monochromator (see Figure 1). The beam size illuminating the sample was 10 mm (*w*) × 1 mm (*h*).  Figure 2 shows the apparatus setup to measure fluorescent yield from the sample. A 10-cm-long ion chamber filled with a gas mixture of N_2_ (30 mbar) and He (983 mbar) was employed to measure intensity of the X-ray beam before the sample. The ion chamber absorbed only 10% of the beam intensity through ionization of the gas filling and produced a small electrical current signal proportional to the beam intensity.

Fluorescence X-rays were emitted by the sample and recorded using a 13-channel Germanium detector (GeD) or a Lytle detector (LyD). The GeD has an advantage over the LyD in its ability to distinguish X-ray fluorescence energy. With the use of a digital window, only K_*α*_ photons from sulfur were counted. The final photon counts were averaged over all the channels. Similar to the ion chamber, the LyD produces small electrical current signal corresponding not only to fluorescence X-rays by the sulfur present, but also for other elements present in the sample and scattered photons from the primary beam. The sample chamber was flooded with Helium gas to reduce X-ray absorption and scattering by air. A thin polypropylene window was used to protect the GeD from Helium gas, which can diffuse through thin detector seal of beryllium spoiling cryostat vacuum of the detector. A minimum air gap of 5 mm was introduced for detector clearance. The distances from the sample to detector were 7 mm and 9 mm for GeD and LyD, respectively. 

XANES spectra were recorded from 2450 eV to 2520 eV with the energy step of 0.2 eV and energy calibrated using the maximum absorption of iron sulfate at 2481.4 eV [18]. Absorbance was calculated by the ratio of photon counts from the fluorescent detector to the ion chamber. All QFF samples were applied to the adhesive side of the polyimide tape and placed at a 45° angle to the beam path in the sample chamber. Without dilution, similar approach was used for the preparation of environmental samples. The data processing and quantitative XANES analyses were conducted using the ATHENA program in the IFEFFIT computer package [19].

#### 2.1.2. Sampling Equipment

Ambient air samples were collected by using a Graseby-Anderson high volume air sampler with TSP and PM_10_ TE-6001, operating at a flow rate of 1.4 m^3^ min^−1^. TSP and PM_10_ samples were collected on 47 mm Whatman quartz microfiber filters (QMFs). The filters were preheated at 800°C for 12 hours prior to sampling. The exposed filters were stored in a refrigerator at about 4°C until sulfur speciation analysis to prevent the evaporation of volatile compounds. Both the field sampling and weighing of filters were performed in compliance with the US EPA's guideline Standard Operating Procedure for sampling and handling of PM_2.5_ filters. Note that all filters were weighed by Mettler Toledo AB204-S Analytical Balance before being sent to Synchrotron Light Research Institute (Public Organization), Thailand.

#### 2.1.3. NIDA Bangkapi Campus Monitoring Site

**Figure 3:**
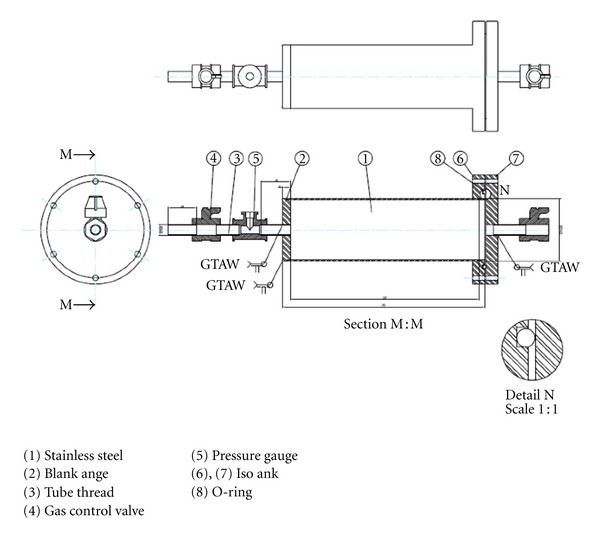
Schematic view of the anoxic preservation stainless cylinder (APSC).

**Figure 4:**
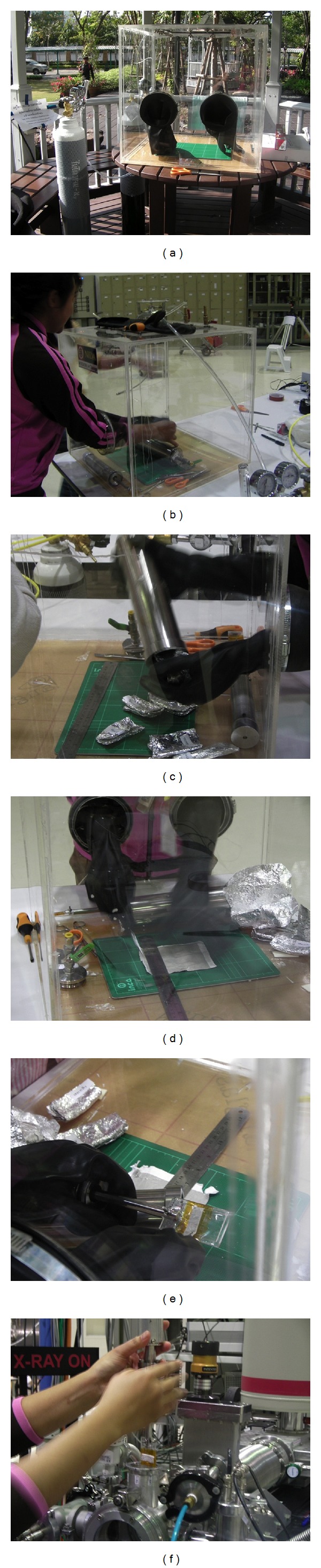
Anoxic preservation processes and instrumentation include (a) pressure controlled glove box (PCGB) for anoxic preservation at sampling site, (b) installation of APSC inside the PCGB, (c) removal of QFFs from APSC, (d) cutting of QFFs, (e) attachment of QFFs to sample holder, and (f) installation of QFFs inside DCM.

PM_10_ samples were collected every three hours over a 27-hour period (i.e., *n* = 9, 18.00–21.00, 21.00–00.00, 00.00–03.00, 03.00–06.00, 06.00–09.00, 09.00–12.00, 12.00–15.00, 15.00–18.00, and 18.00–21.00) at the National Institute of Development Administration (NIDA), Thailand's most prestigious graduate university under the Commission for Higher Education, the Ministry of Education in Bangkok. Diurnal variation of urban baseline samples was taken in an open field located at the south-western side of NIDA's Bangkapi campus, close to Sereethai Road, one of the most heavily used roads in this area. Since the monitoring site is only 1 km away from the Mall Shopping Center Bangkapi, Tesco-Lotus and Makro Stores, it seems reasonable to assume that PM_10_ collected from this site represent aerosols derived from traffic vehicle exhausts. As previously mentioned, all samples were gathered on Whatman QM-A-type quartz fiber filters (QFFs), and carefully preserved inside a Pressure Controlled Glove Box (PCGB) purged with nitrogen gas to avoid conversion of gaseous SO_2_ to particulate SO_4_^−2^. All filters were installed inside an Anoxic Preservation Stainless Cylinder (APSC) filled with nitrogen gas (see Figures 3 and 4) and carefully delivered to Synchrotron Light Research Institute (SLRI) for sulfur k-edge XANES spectra analysis.

#### 2.1.4. Source Cluster Monitoring Sites

Sampling was conducted over three consecutive days at (Prince of Songkla University (PSU), Hat-Yai Campus), Traffic Intersection (TI), Corps Incinerator (CI), Charoen Phokphand Factory (CPF), Songkla Lake (SL), Rubber Manufacturing Factory (RMF), Bus Terminal (BT), Waste Incinerator (WI), Barbeque Festival (BF), Petkrasem Road (PR) and Kor Hong Hill (KHH) stations. The air samples collected at BB and PTB stations represent the most important in terms of air quality. Therefore, 3-hour samples were collected three times per day in order to avoid any overloading of air particulate matter at these three stations. 

All monitoring sites are located in Songkhla province, which is situated 950 km south of Bangkok with a population of over 1.32 million people. Thirteen sampling locations were carefully selected to reflect the complex urban environment a mixture of commercial, residential, and industrial estates that are present in this province. Sampling locations were selected and categorized by the nature of emission sources. The sites descriptions are given below in Table 1.

**Cluster I**

The baseline sampling site cluster was further categorized into three groups.

**Prince of Songkla University (PSU)**

The site is situated about 3 m above ground level in the Faculty of Environmental Management, Prince of Songkla University, and about 550 m away from the main road that leads to the city center of Hat-Yai. It is important to note that PSU represents the sampling period of October (24th–26th October, 2007). This site is considered an urban residential zone.

**Songkhla Lake Monitoring Station (SL)**

It is situated about 13 km from the northern side of Prince of Songkla University, south of Songkhla Lake, and about 14 km from the western side of the Gulf of Thailand. This sampling station is far from any industrial and traffic emission sources, including chemical and metallurgy, and power plants in this district. Therefore it is used as the *rural baseline* monitoring station. Songkla 1 (SL1) and Songkla 2 (SL2) were used to represent the monitoring periods of July (from 27th to 29th July, 2007) and October (from 20th to 22nd October, 2007) respectively.

**Korhong Hill Monitoring Stations (KHH)**

It is located on the top of Kor-Hong hill with an elevation of 356 meters. This site represents a mixture of all emission sources in the urban area. Therefore, the air mass collected from this station was considered the Urban Baseline. The sampling was conducted on 3rd to 5th November, 2007.

**Cluster II**

This group contains sampling sites which are categorized as a source of diesel and benzene emissions.

Three sampling stations were classified in this cluster.

**Bus Terminal (BT)**

This site is located at the south-western side of PSU and approximately 1.4 km from the campus. As the majority of buses are diesel fuelled in this area, we considered this site a source of diesel emissions. The air sample collection was conducted from 5th to 7th August 2007.

**Petkrasam Road (PR)**

This site was located in the heart of the city of Hat-Yai. This site faces the heaviest burden of traffic congestion with a mixture of diesel and gasoline exhaust emissions. Thus, the air mass collected from this area is mainly contaminated with diesel and benzene emissions. The monitoring was conducted on 27th–29th August, 2007.

**Traffic Intersection (TI)**

This station is located at the traffic intersection in front of the main gate of PSU near a Tesco supermarket. It is on the eastern side and approximately 2.5 km from the Hat-Yai city center close to an urban residential zone. This station is regarded as a heavy traffic area and mainly contaminated with diesel and benzene emissions. The air samples were collected from 5th to 7th July, 2007.

**Cluster III**

This cluster contains sites which are categorized as a source of industrial emissions. Two sampling stations were classified in this cluster.

**Charoen Phokphand Factory (CP)**

This site is located at the CP fish cannery. This factory is part of the largest business conglomerate in Thailand. We considered it a source of crude oil-burning emissions. The monitoring was conducted from 24th to 26th July 2007.

**Rubber Sheet Manufacturing Factory (RMF1 and RMF2)**

This station is located at Tumbol Tungwan, Hat-Yai district. This factory normally uses para rubber treatment as fuel for the manufacturing process. The rubber sheets are treated with high-temperature and high-pressure steam and then purified with sulfuric acid solution. As para rubber trees are used as fuel for this process, we therefore considered it a source of mixed para rubber tree burning emissions, with latex particles and sulfuric acid aerosols also expected. The air samples were collected from 30th July to 1st August 2007 (RMF1) and from 2nd to 4th August 2007 (RMF2).

**Cluster IV**

This group contains sampling sites classified as biomass burning sites. We further categorized the sites into two subgroups as follows.

**Para Rubber Tree Burning (PTB)**

This station is located at Namom district, Songkhla Province and can be recognized as a source of Para rubber tree burning emissions. The air samples were collected on 18th November, 2007.

**Biomass Burning (BB)**

This station is located in a rice field in Satingpra district, Songkhla Province. Removing rice straw left after the crop is harvested by burning can limit disease organisms but leads to serious air pollution problems in this region. This station was considered a source of biomass burning emissions. The sampling was conducted on 16th November, 2007.

**Cluster V**

This cluster is classified as old tire, clinical waste and charcoal burning sites. We further categorized the sites into the following three groups.

**Corpse Incinerator (CI)**

This station is a part of Kor-Hong temple, located on the northern side and about 1.5 km from TI. Since timber and tires are generally used as fuel for corpse incineration, this site is considered an emission source of both timber and tire burning. This site represents the sampling period of 19th–21st July 2007.

**Waste Incinerator (WI)**

This site is situated in the city center and belongs to the municipality of the city of Hat-Yai. Since the municipal waste incinerated is a heterogeneous mixture of solid waste and burnt fuels, this site can be recognized as a source of a combination of solid waste burning and diesel exhaust emissions. The air samples were collected from 28th to 30th August, 2007.

**Barbeque Festival (BF)**

This site is located on the PSU campus on the top roof of the Faculty of Natural Resources building. The barbeque festival has become an annual tradition that is held on the second week in August. The 40th Annual Barbecue Festival was on Wednesday, August 15th, 2007. This site can be considered as an emission of charcoal burning emissions. The air samples were collected from 15th to18th August, 2007. Additionally, it is also important to emphasize that there were no obstructions in the vicinity of the sampling equipment at all sites, which were strategically positioned to be accessible to winds from all directions.

#### 2.1.5. Vertical Sampling Sites

All three sampling sites are located in the city centers of Bangkok, Chiang-Mai and Hat-Yai, representing the country's capital and largest city alongside the largest cities in the northern and southern regions of Thailand, respectively. Air samples were collected at three different heights for three hour periods (i.e., 9.00 am–12.00 am) at Bai-Yoke Suite Hotel Observatory Site (i.e., Level-1: 38 m; Level-2: 158 m; Level-3: 328 m above ground level), at Centara Duangtawan Hotel Observatory Site (i.e., Level-1: 12 m; Level-2: 52 m; Level-3: 152 m above ground level) and at Novotel Centara Hat-Yai Hotel Observatory Site (i.e., Level-1: 30 m; Level-2: 60 m; Level-3: 125 m above ground level) as monitoring sites for Bangkok, Chiang-Mai and Hat-Yai in that order (see Table 1). 

While traffic emissions are considered the main source of air pollutants in the Bangkok atmosphere, agricultural burning is the major air pollution problem in Chiang-Mai. A high concentration of traffic emission residue is expected for Bangkok samples, while samples from Chiang Mai will likely contain evidence of industrial emissions and wood burning. Hat-Yai is located only 30 km from the west coast of the Gulf of Thailand, so it is likely that aerosol samples from this site will contain traces of sea salt and biomass burning.

## 3. Results 

**Figure 5:**
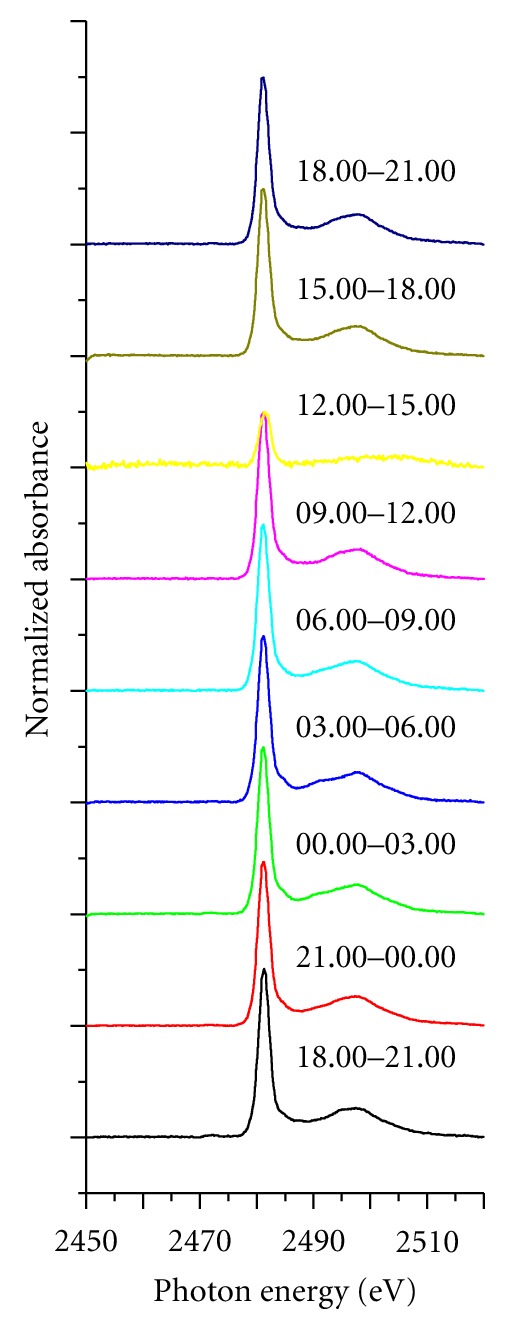
Sulfur K-edge XANES spectrum of urban PM_10_ collected at NIDA, located in Bangkok.

Figure 5 illustrates the diurnal variation of nine sulfur K-edge XANES spectra of PM_10_ preserved in APSC and PCGB filled with nitrogen gas prior to XANES analysis at SLRI. There are three features which are common to each sulfur K-edge XANES spectrum: (a) the “pre-edge” region, which represents the energy emitted at 2450–2465 eV and points to the area of energies lower than the main absorption edge; (b) the “near-edge” region, which refers to the 5 eV wide region around the white-line (i.e., the main peak), located at the “post-edge” region, which denotes the region greater than 2490 eV outside the absorption edge. Based on the results from nine sulfur K-edge XANES spectra, one can deduce that all S species in PM_10_ share comparable spectral structures. PM_10_ samples all seem to show a relatively flat spectrum of preedge region (i.e., 2450–2650 eV), followed by a sharp main peak at 2482 eV, and a large postedge fluctuation that arises in the energy band of 2490–2510 eV (see Figure 5). These results are consistent with previous reports focusing on sulfur K-edge XANES spectra of aerosols with different particle sizes [11–13].

Drawing from the Takahashi et al. conclusion that particulate sulfur compounds are dominated by S(VI), it can be deduced that the oxidation number of elemental sulfur collected at NIDA Bangkapi campus is also +6, and plausibly occupied by sulfate species. 

**Figure 6:**
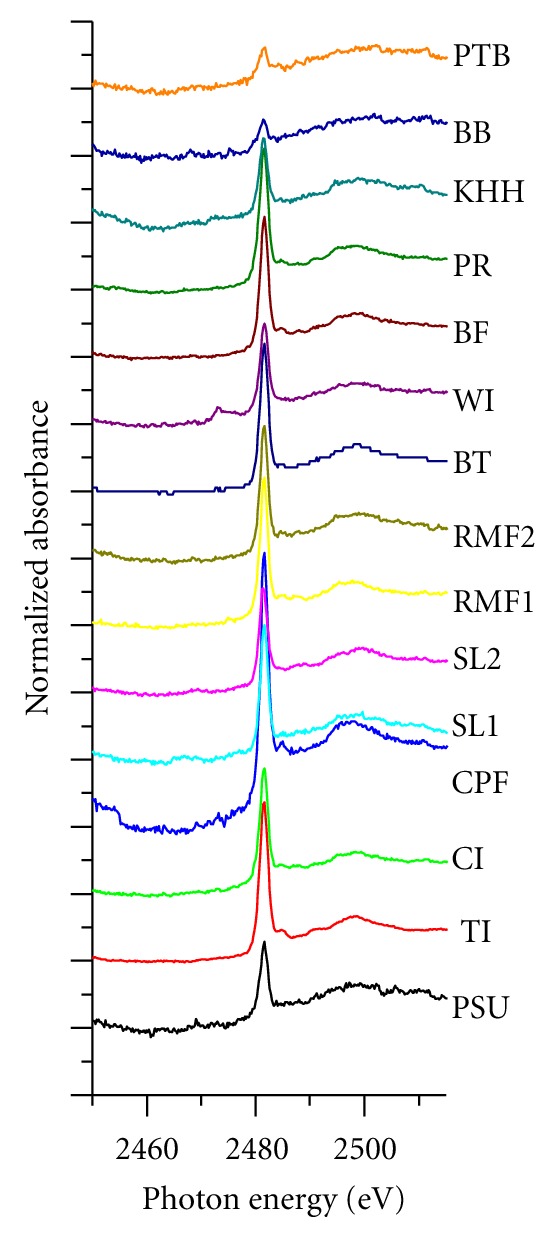
Comparison of sulfur K-edge XANES spectra of PM_10_ obtained from various emission sources in Songkla province.

Figure 6 demonstrates the sulfur K-edge XANES spectra of 15 PM_10_ samples collected from various emission sources in Songkla province. Similar to the previous sulfur K-edge XANES spectra detected at NIDA Bangkapi campus, the spectra from BT, PR, Tesco, RMF, and CPF display a broad resonance at the preedge region, probably due to a superimposition of more than one resonance. For all 15 spectra, a maximum peak appears at the higher energy of 2482 eV, which is consistent with peaks belonging to diurnal variation samples. Despite several deviations in the intensities of the maximum peak between the different emission source samples, some other subtle dissimilarities in post-edge regions are apparent, particularly in the subsequent structure, which appears the post-edge region between 2485 and 2510 eV. Previous studies interpreted this phenomenon as a consequence of electron transitions with a d-type shape resonance, which can be influenced by various types of cat ions binding with sulfates [20, 21]. These discrepancies among different sulfur K-edge XANES spectra can be taken to characterize the “finger print” of target aerosols associated with various emission baselines.

Figure 8 underscores the dominance of the sulfate species in urban aerosols by displaying the relatively similar sulfur K-edge XANES spectra of PM_10_ collected at different heights for comparison to “diurnal variation” samples. The analogous spectral features were observed in PM_10_ collected at Chiang-Mai, Bangkok and Hat-Yai, irrespective of differences in sampling height. Based on the positive correlation between t sulfur and photon energy, it appears sensible to consider the highest peak structures at 2482 eV as an outcome of the majority of S(VI) recorded in urban PM_10_. It is also plausible to assume that ammonium sulfate and gypsum are prime candidates based on the systematics of this region as mentioned in previous study [8, 11, 20]. 

In addition, earlier studies revealed that the molecular structure has little effects 1s → 3p transition energy of that specific sulfur functional group, thus highlighting the usefulness of XANES to categorize the oxidation number of elemental sulfur in PM_10_ [22–24].

## 4. Discussion

### 4.1. Effect of “Anoxic Preservation” on Sulfur K-Edge XANES Spectra of PM_10_

To investigate the “anoxic preservation” effect on sulfur K-edge XANES spectra of PM_10_, QFF samples were preserved in APSC filled with nitrogen gas immediately after completing collections. It is also important to note that all filter-cutting processes were conducted inside PCGB filled with nitrogen gas prior to XANES analysis. 

The sulfur K-edge XANES spectra of PM_10_ samples collected at different periods demonstrated that the position of the main peak was relatively consistent at 2482 eV without any sign of peak shift within ±0.5 eV for numerous aerosol samples, indicating that particulate sulfur compositions are not influenced by diurnal variation. The moderately uniform sulfur K-edge XANES spectra suggest that the overall sulfate species distribution is constant at the traffic site. This finding is consistent with similar works performed by Takahashi et al., indicating that “anoxic preservation” might have minor importance for the oscillation of sulfur K-edge XANES spectra in PM_10 _[11–13]. 

Three interpretations can be considered for this phenomenon. Firstly, all sulfur compounds from emission sources in urban atmospheric environment are composed of S(VI) and conceivably predominated by sulfate species. Secondly, the oxidation rate of aerosol sulfur is too fast to convert all particulate sulfur compounds to the most stable oxidation state of +6 before being transported to the receptor site at NIDA Bangkapi campus. 

Thirdly, all sulfur compounds were completely oxidized to S(VI) after the deposition of particles on QFFs within three hours of sampling period. Interestingly, this finding is in good agreement with an earlier study, which applied XANES for the determination and quantification of S(IV) species in aerosol samples collected in Qingdao, northeastern China [13]. Higashi et al. concluded that sulfite was the only sulfur which possessed the oxidation number of +4 detected in aerosols, particularly in particles with larger diameters collected in August 2001.

 It is also crucial to stress that no S(IV) species, however, were detected at the surface of the aerosols as demonstrated by surface-sensitive conversion electron/He ion yield XANES [13]. In the case of PM_10_ collected at NIDA Bangkapi campus, the moderately high reactive trace gas (e.g., NO_*x*_, O_3_, and OH radical) concentration released from diesel engines and other traffic combustions could oxidize sulfur compounds and conclusively convert all of the sulfur oxidation states to S(VI). In addition, the relatively low absorption intensity of sulfur K-edge XANES spectrum of PM_10_ collected from 12.00 am to 15.00 pm can be described as a consequence of the “dilution effect” triggered by the lifting of warm air mass at midday (see Figure 5).

### 4.2. Effect of “Emission Sources” on Sulfur K-Edge XANES Spectra of PM_10_

Unlike the results of diurnal variation campaign, the comparatively oscillating post-edge regions were discernible and thus highlighted the intricacy of sulfate compositions in PM_10_ collected from various emission sources (Figure 6). Likewise, the white-line intensity fluctuations are more noticeable in emission source spectra. These unique spectra features can be diagnosed as a divergence in both “amount” and “speciation” of S detected in PM_10_. Interestingly, the unsmooth pre-edge regions were predominant in sulfur K-edge XANES spectra of CI, WI, CPF, PSU, BB, and PTB (Figure 6). This can be ascribed to both the mixture of source signals (i.e., more variety of sulfur oxidation states) and the relatively low signal-to-noise ratio of S(VI) particularly in case of PTB and BB (i.e., low white line intensities due to high noise Baselines). 

It is also worth mentioning that different sulfur compounds possess distinctive energy white line positions. For example, elemental S, organic polysulfide, thiol, sulfoxide, sulfite, sulfone, sulfonate, ester sulfate, and inorganic sulfate display the peak energy of sulfur K-edge white lines at 2472.5, 2473.0, 2473.4, 2475.8, 2478.7, 2480.2, 2481.3, 2482.5, and 2482.5 eV, respectively [25]. Therefore, the strong oscillation at the pre-edge reveals the crucial information about various sulfur species in target samples [9].

**Figure 7:**
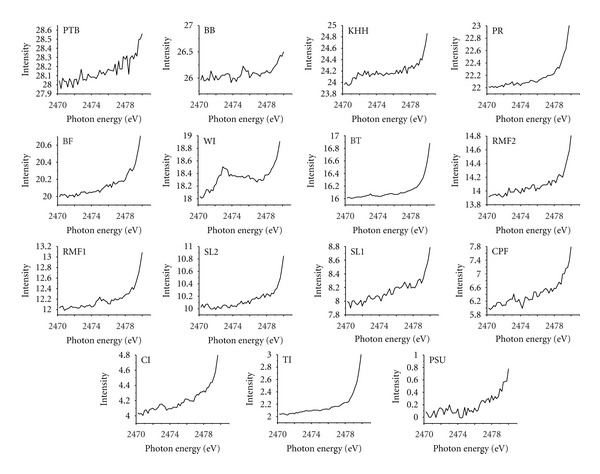
Comparison of sulfur K-edge XANES of PM_10_ collected at various emission sources between 2,470–2,480 eV.

A further investigation of sulfur K-edge XANES spectral features was conducted for baseline samples (i.e., Cluster I). Both SL1 and SL2 showed a considerably high level of reproducibility of pre-edges from 2470 eV to 2480 eV regardless of different sampling periods (see Figures 6 and 7). This pattern of behavior supports the idea that both anthropogenic emissions and seasonal effects played a minor role at this monitoring station due to its distance from the urban district of Hat-Yai city combined with “the dilution effect” triggered by clean maritime air mass coming in from the Gulf of Thailand. 

In contrast, the spectral plateau in the region of 2473 eV–2476 eV detected in WI illustrates an exceptional feature, which cannot be explained by the differences in climatic conditions due to the relatively similar sampling period and location (see Figure 7 and Table 1). The most convincing explanation, therefore, plausibly attributed this difference to the complex nature of solid waste materials combusted in WI. In addition, the comparatively analogous spectral features of PSU, PTB, RMF1, and RMF2 indicated that para rubber tree combustion might play a major role in governing PM_10_ concentrations in residential zones, and thus raising concerns about the adverse impacts of para rubber industry on air quality in the city of Hat-Yai. 

### 4.3. Effect of “Vertical Distribution” on Sulfur K-Edge XANES Spectra of PM_10_

**Figure 8:**
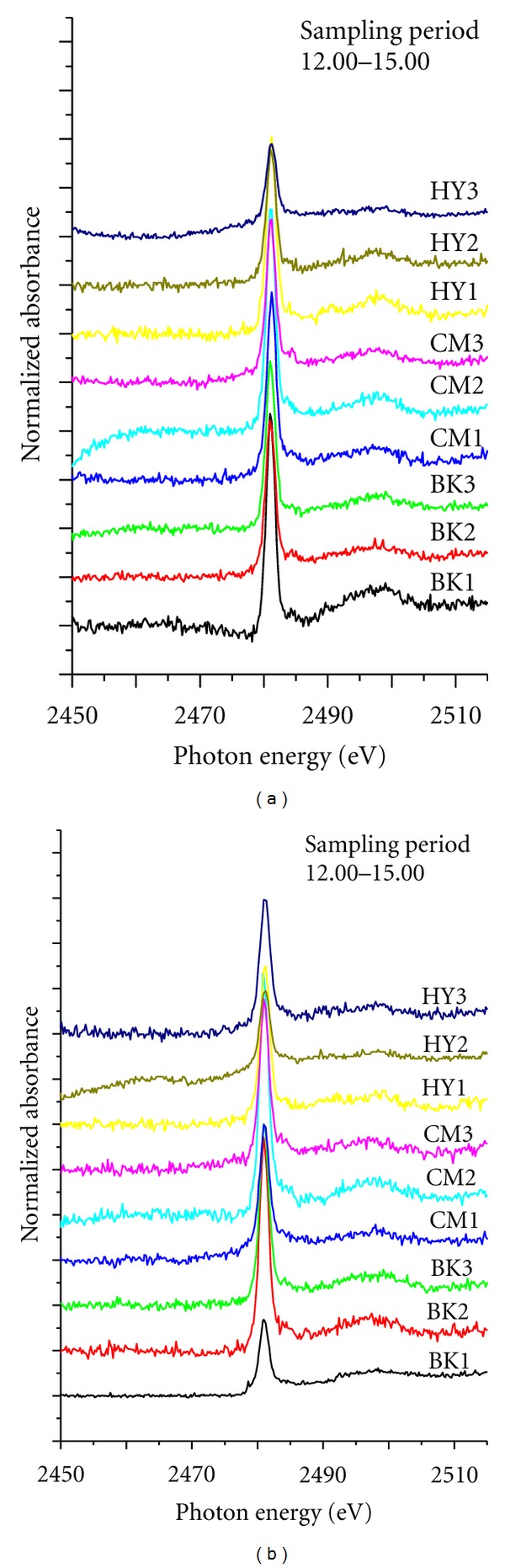
Sulfur K-edge XANES spectra of urban PM_10_ collected at three different altitudes in Bangkok (BK), Chiang-Mai (CM) and Hat-Yai (HY).

To the best of our knowledge, this is the first study to examine sulfur K-edge XANES spectra of PM_10_ at different altitudes under same urban atmospheric conditions. All PM_10_ collected from three different cities displayed moderately similar spectral features in comparison with those of diurnal variation samples monitored at NIDA Bangkapi campus as illustrated in Figure 5. The relatively high daytime air temperature may be responsible for the upwelling of particles, which can be triggered by the convective heat transfer of air mass from surface boundary layer (SBL) to urban boundary layer (UBL). This phenomenon allowed air mass, which is relatively homogeneous vertically, to dominate the urban boundary layer in Bangkok, Chiang-Mai and Hat-Yai during the observations. Despite differences in both “emission sources” and “climatic conditions” among the three cities, all spectral features were consistently identical with the principal composition of S(VI) as illustrated in Figure 8. 

## 5. Conclusions

Several concerns were addressed with respect to factors affecting the sulfur K-edge XANES spectra of PM_10_ collected from various locations with different sampling altitudes. There are three common features that appear across all sulfur K-edge XANES spectra: (a) the “Pre-edge” region, involving the energy of 2450–2465 eV, which signifies the area at energies lower than the main point of the absorption edge, (b) the “Near-edge” region, which refers to the 5 eV-wide region immediately encompassing the white-line region (i.e., the main peak), which is located at 2482 eV, and (c) the “Post-edge” region, which denotes the region over 2490 eV, outside the absorption edge. Neither “anoxic preservation” nor “emission source” appreciably changed this pattern. Further investigation was conducted to assess the alteration of particulate sulfur K-edge XANES spectra as a function of sampling altitude. Interestingly, comparatively uniform sulfur K-edge XANES spectra were observed in all PM_10_ collected from different cities and sampling periods. This study also ascertains that the sulfur oxidation state of PM_10_ is predominantly overwhelmed by S(VI).

## Figures and Tables

**Table 1:** Descriptions of sample type, duration, and location.

Site	Source type	Sampling period	Latitude				Longitude			
PSU	Urban residential zone	24/10/07–26/10/07	7°	00′	21.28′′	N	100°	29′	53.27′′	E
TI	Traffic	05/07/07–07/07/07	7°	00′	30.81′′	N	100°	29′	39.21′′	E
CI	Timber	19/07/07–21/07/07	7°	01′	17.55′′	N	100°	29′	41.44′′	E
CPF	Crude oil	24/07/07–26/07/07	6°	54′	16.38′′	N	100°	28′	05.15′′	E
SL1	Rural baseline	27/07/07–29/07/07	7°	10′	02.92′′	N	100°	35′	11.36′′	E
SL2	Rural baseline	20/10/07–22/10/07	7°	10′	02.92′′	N	100°	35′	11.36′′	E
RMF1	Timber	30/07/07–01/08/07	7°	03′	19.97′′	N	100°	37′	58.90′′	E
RMF2	Timber	02/08/07–04/08/07	7°	03′	06.28′′	N	100°	24′	07.77′′	E
BT	Diesel engine	05/08/07–07/08/07	6°	59′	42.78′′	N	100°	28′	58.02′′	E
WI	Solid waste + crude oil	08/08/07–10/08/08	6°	57′	15.43′′	N	100°	24′	00.46′′	E
BF	Charcoal	15/08/07–17/08/07	7°	00′	23.09′′	N	100°	30′	00.54′′	E
PR	Traffic	27/08/07–29/08/07	7°	00′	52.99′′	N	100°	28′	20.50′′	E
KHH	Urban baseline	03/11/07–05/11/07	7°	00′	57.92′′	N	100°	31′	12.76′′	E
BB	Biomass burning	17/11/07	6°	57′	40.45′′	N	100°	33′	06.68′′	E
PTB	Para rubber tree burning	18/11/07	6°	57′	40.45′′	N	100°	33′	06.68′′	E
BKK-L1	Urban baseline	18/02/08	13°	45′	46.30′′	N	100°	32′	25.37′′	E
BKK-L2	Urban baseline	18/02/08	13°	45′	46.30′′	N	100°	32′	25.37′′	E
BKK-L3	Urban baseline	18/02/08	13°	45′	46.30′′	N	100°	32′	25.37′′	E
CM-L1	Urban baseline	25/02/08	18°	47′	02.76′′	N	98°	59′	56.40′′	E
CM-L2	Urban baseline	25/02/08	18°	47′	02.76′′	N	98°	59′	56.40′′	E
CM-L3	Urban baseline	25/02/08	18°	47′	02.76′′	N	98°	59′	56.40′′	E
HY-L1	Urban baseline	17/12/07	7°	00′	20.29′′	N	100°	28′	16.44′′	E
HY-L2	Urban baseline	17/12/07	7°	00′	20.29′′	N	100°	28′	16.44′′	E
